# Why men age faster but reproduce longer than women:  mTOR and evolutionary perspectives

**DOI:** 10.18632/aging.100149

**Published:** 2010-05-15

**Authors:** Mikhail V. Blagosklonny

**Affiliations:** Department of Cell Stress Biology, Roswell Park Cancer Institute, Buffalo, NY 14263, USA

**Keywords:** aging, reproduction, longevity, lifespan, rapamycin, menopause

## Abstract

Women live longer than men. Yet, it is believed that men do not age faster than women but simply are weaker at every age. In contrast, I discuss that men age faster. From evolutionary perspective, high accidental death rate in young males is compatible with fast aging. Mechanistically, hyper-activated mTOR (Target of Rapamycin) may render young males robust at the cost of accelerated aging. But if women age slower, why then is it women who have menopause? Some believe that menopause is programmed and purposeful (grandmother theory). In contrast, I discuss how menopause is not programmed but rather is an aimless continuation of the same program that initially starts reproduction at puberty. This quasi-program causes over-activation of female reproductive system, which is very vulnerable to over-activation. Mechanisms of aging and menopause are discussed.

## Longevity: men and women

Women have lived longer than men in different countries and in every era [1]. In 1980 in the USA, the estimated life expectancy at birth was 70 years for men and 77.5 years for women [2]. In the world, 75% and 90% of people older than 100 years and 110 years (respectively) are women. And the longest living person (122 years old) was a woman. But do women age slower than men? The conventional opinion is that women and men age at the same rate but men are ‘less robust' than women [1]. Seemingly in agreement, the mortality rate is lower in young women compared with young men. In women, the mortality rate is lower at every age, even in childhood. In other words, "women do not live longer than men because they age slowly, but because they are more robust at every age" [1]. This reasoning would be correct if causes of death were the same at every age. However, young and old men die from different causes. Young men die from accidents, while old men die from aging (technically speaking, from age-related diseases).

## High accidental death rate and fast aging (evolutionary perspective)

There is a very noticeable jump of mortality in the late teens in men [1]. Young men are often engaged in competitive, reckless, and dangerous activities. Therefore, even in modern society, the accidental death rate is high in young men. Historically, the accidental death rate in men was much higher than it is now. (Due to a fierce competition for status and mates, due to fights and wars, young men were killed at a very high rate). So, historically, men had lower chances to survive into old age than women had. And, according to evolutionary theory, a high accidental death rate determines fast aging [3-5]. If most men died young from accidental death, then they could not live long enough to experience aging. Then there was no natural selection to postpone aging. So accelerated aging in men is predictable from evolutionary perspective. But accelerated aging is also predictable mechanistically.

## Mechanistic explanation: antagonistic pleiotropy and mTOR

In males, muscle hypertrophy and heavy body helps to compete with other males. (In fact, men are larger than women.)  Cellular growth and hypertrophy are stimulated by the mTOR (mammalian Target of Rapamycin) intracellular signaling pathway. Insulin, growth factors, amino acids, glucose lipoproteins, and testosterone all activate the mTOR pathway [6-9].   In turn, the mTOR pathway stimulates protein synthesis and cell size growth [10]. For example, skeletal muscle hypertrophy depends on the mTOR pathway [11,12]. In addition, inhibition of the mTOR pathway decreases testosterone levels and spermatogenesis [13]. Thus, activation of mTOR may provide a selective advantage to young males.

On the other hand, the mTOR pathway is required forcellular senescence of mammalian cells [14-18]. Cellular aging is driven by the remaining activation of mitogenic signaling pathways in post-mitotic cells [19,20]. In fact, mechanistically, aging is a continuation of growth, driven in part by mTOR [21].  In agreement, mTOR is involved in age-related diseases such as atherosclerosis, neurodegeneration, cancer, which are deadly manifestations of aging (see for review [22-24]). And rapamycin prolongs lifespan in mammals [25].

**Figure 1.**
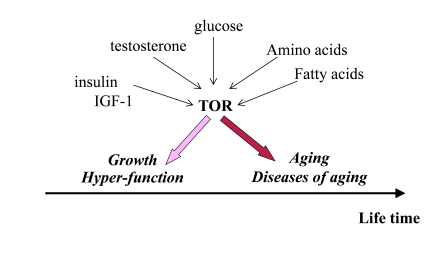
Program of growth and quasi-program of aging. The TOR pathway is activated by growth factors, hormones and nutrients. This activation is beneficial early in life by stimulating growth and muscle hypertrophy. Evolutionary perspective: This was especially important for prehistoric men, living in dangerous environment that required physical strength.  mTOR is involved in aging later in life, but most men died young from accidental death. Thus, robustness early in life is associated with accelerated aging.

Thus, over-activation of mTOR may provide an advantage (muscle hypertrophy, high levels of testosterone and high spermatogenesis) in early life at the cost of accelerated aging later in life. As an illuminating example, mice over-expressing growth hormone exhibit increased levels of IGF-I and adult body size, reduced life span and reproductive life span [26]. (Note: IGF-I stimulates mTOR, Figure 1).

## Accelerated age-related diseases in men

Humans do not die from "healthy" aging. Humans die from age-related diseases. The mTOR pathway is involved in age-related diseases such as cancer, atherosclerosis, hypertension, heart failure, osteoporosis, type II diabetes [22,24,27]. These diseases are deadly manifestations of aging. When aging is accelerated, age-related diseases occur earlier in life too. Healthy aging (a late onset of diseases) is associated with longevity (see for discussion [28]). For example, centenarians (100 years old or older) show a delay in the onset of age-related diseases, including cardiovascular disease, type 2 diabetes, cancer and Alzheimer's disease. In other words, those who age slower are healthier [29,30].

If women age slower than men, then age-related diseases must be delayed in women. In fact, most age-related diseases are delayed in women compared with men. For example, coronary atherosclerosis is postponed in women. Not only atherosclerosis, but also cancer and most other diseases of aging occur earlier in men than in women [31]. Women also live more years than men free of each of these diseases with the exception of arthritis [32]. Women rarely die from age-related diseases before menopause. The later onset of diseases in women compared with men suggests that women age slower than men.

Intriguingly, slower erosion of human telomeres favor females [33] and, even further, the rate of leukocyte telomere shortening predicts mortality from cardiovascular disease in elderly men [34]. I speculate that high rate of telomere shortening reflects cellular hyper-activation and may be suppressed by rapamycin.

## Aging *versus* reproductive aging

Yet common wisdom holds that women age faster than men. One should not confuse aging and subjective perception of youthfulness and sexual attractiveness, which reflects fertility. Aging is an increase of the probability of death. And a 50-year-old man has higher chances to die than a 50- year-old woman. Furthermore, men acquire grey hair and wrinkles faster than women and thus men even ‘look' older [35]. Although men age faster, they can reproduce longer. And here is another puzzle: why women undergo menopause.

Like aging itself, menopause is tolerated by natural selection, because women (until recently) did not live long enough to experience it. (In modern society, there must be a very strong natural selection for delayed menopause). So an evolutionary explanation is simple: ancestral women did not live long enough to have menopause. But male lifespan was even shorter: why then do men not have menopause? What is so special about female reproduction?

## Can menopause be programmed?

There is common opinion among traditional gerontologists that menopause is beneficial for women, has an evolutionary advantage and is adaptive [36-38]. It was suggested, for instance, that menopause prevents death of women in labor. The most popular is a "grandmother hypothesis" that menopause frees older women to help their daughters to raise grandchildren. This is a sort of group-selection hypothesis. Why do not daughters delay reproduction just in order to help their mothers raise siblings?  Or what is the biological sense to stop reproduction, if a woman has no grandchildren living with her? The crucial assumption of ‘grandmother' hypothesis is that menopause occurs only in humans [37]. Yet, menopause was documented in non-human primates, rodents, whales, dogs, rabbits, elephants and domestic livestock [39]. It was shown, for instance, that mice eventually undergo ovarian changes analogous to menopause in humans [40,41].

 It was shown that grandmothers may promote survival of their maternal grandchildren in Gambia [37]. Grandmothers are useful but menopause is not. There is no experimental evidence that menopause is beneficial even when women live with grandchildren in Gambia. Menopause accelerates age-related diseases such as atherosclerosis, osteoporosis and cancer [42,43]. Reproductive death provides no selective benefit (unless group-selection theories of aging are correct) and ‘grandmother hypothesis' contradicts the evolutionary theory. If aging is not programmed, then reproductive aging is not programmed too.

**Figure 2.**
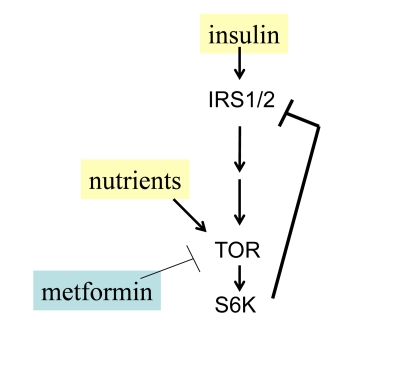
Negative feedback and insulin resistance. TOR is activated by nutrients and insulin and in turn causes depletion of IRS1/2 and insulin resistance. Whereas nutrients activate TOR, low nutrients and metformin deactivate TOR.

## TOR-driven quasi-programmed aging

Aging is not programmed but quasi-programmed [22,44-46]. ("Quasi-" means  "as if, resembling"). Quasi-program is an aimless continuation of a useful program that was not switched off upon its completion. Unlike a program, a quasi-program has no purpose. Developmental programs become aimless quasi-programs later in life. Quasi-programs are driven by antagonistic pleiotropic genes, which are beneficial early in life on the cost of aging later in life. Most genes that control aging and longevity constitute the mTOR pathway [22,23]. mTOR is absolutely essential during embryonic development [47,48]. In post-development, mTOR is involved in aging and age-related diseases [22].

Nutrients activate mTOR and cause insulin-resistance in cell culture [49,50] as well as systemically in rodents and humans [51-54]. There is a negative feedback loop between insulin signaling and TOR (Figure 2). When mTOR is activated, it blocks insulin signaling (insulin resistance) [49,55]. Noteworthy, insulin resistance is associated with premature menopause in some patients [56].

## The menstrual cycle is fragile

**Figure 3.**
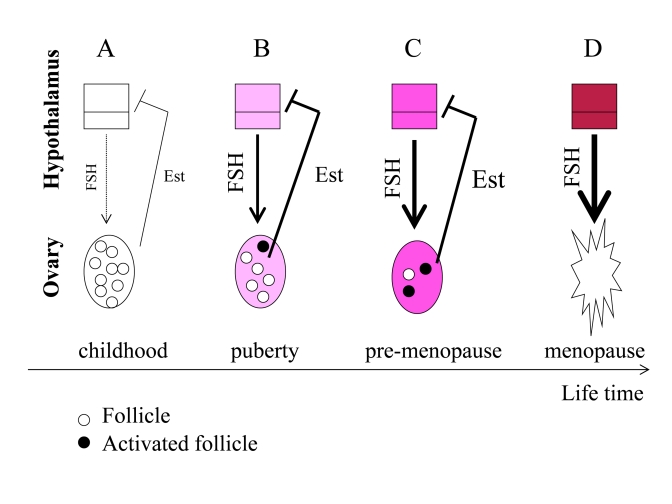
From programmed puberty to quasi-programmed menopause. For simplicity, only the FSH-estrogen feedback loop is shown. FSH stimulates follicles and production of estrogens (Est). Estrogens inhibit FSH production (negative feedback). (**A**) In girls, the hypothalamus is extremely sensitive to estrogens and even low levels of estrogens inhibit FSH. (**B**) The onset of menstrual cycle. While the hypothalamus is becoming resistant to estrogens, FSH stimulates the ovaries and estrogen production. Progressive activation of follicles from the dormant pool serves as the source of fertilizable ova. (**C**) Pre-menopause. While the hypothalamus is becoming progressively resistant to estrogens, FSH progressively over-stimulates the ovaries. (**D**) The ovaries fail. Menopause occurs when the primordial follicle pool is exhausted. Estrogen levels drop. The feedback between hypothalamus and the ovaries is disrupted.

Since aging is not programmed, it does not hurt on purpose. It does not cause ovarian failure (menopause) on purpose. The logic of aging is simple: the most fragile systems fail first. A female reproductive system is fragile because it depends on exact interactions between The hypothalamus and ovaries, communicating via dozens of hormones. The menstrual cycle is regulated by interplay of negative and positive feedback loops. The hypothalamus stimulates the pituitary gland to secrete Follicle-Stimulating Hormone (FSH), which in turn stimulates follicles in the ovaries (Figure 3). Follicles maturate and secrete estrogens. Estrogens inhibit the hypothalamus, decreasing secretion of FSH (a negative feedback loop).  In turn, FSH stimulates ovarian follicles, which produce estrogens, which in turn inhibit FSH production. Also, estrogens stimulate secretion of Lutenizing Hormone (LH). LH in turn causes ovulation. So for the normal menstrual cycle, the hypothalamus should have a narrow range of sensitivity to estrogens. Both too high and too low sensitivities are not compatible with menstrual cycles. In comparison, regulation of reproduction in men is simpler. There is a gradual decrease in fertility in men too (analogous to pre-menopause), although this usually does not result in testicular failure during a man's lifetime [57,58].

## Quasi-programmed menopause

A half century ago, Vladimir Dilman proposed a "biological clock" that initially launches reproduction in puberty and then causes menopause [59,60]. This idea is absolutely compatible with quasi-programmed nature of menopause, as discussed herein.

Before puberty, the hypothalamus is extremely sensitive to estrogens (Figure 3 A). Even low levels of estrogens suppress FSH production and, therefore, levels of FSH are low. At puberty, the hypothalamus becomes more resistant to estrogens. Then low levels of estrogens cannot suppress FSH. FSH in turn stimulates the ovarian follicles. Follicles produce estrogens, which in turn inhibit FSH production (Figure 3 B). During lifetime, resistance to estrogens continues to increase (Figure 3C). This ever-increasing resistance is an aim-less continuation of the same program that initiated menstrual cycle at puberty.  FSH is elevated in pre-menopause and rising serum FSH levels is one of the earliest signs of human female reproductive aging [61], [62]. Rising FSH levels over-stimulate the ovaries (Figure 3C), thus depleting follicles (Figure 3D).

FSH hyper-stimulates the ovaries, causing more follicles to be recruited simultaneously (Figure 3 C). This may explain the increased tendency of older mothers to have dizygotic twins [63]. Due to hypothalamic resistance to estrogens, estrogens cannot induce LH surges, which are necessary for ovulation. Therefore, follicles are recruited without progression to ovulation. Therefore, fertility gradually decreases long before menopause.

Hypothalamic resistance to estrogens causes higher FSH levels and lower LH pulses, disturbed feedback relationships and decrease in fertility [64]. Levels of estrogens tend to be increased in pre-menopause [64], but even increased estrogens cannot suppress FSH [61]. FSH over-stimulates follicle recruitment, leading eventually to follicular depletion (Figure 3D). This process eventually results in ovarian failure (Figure 3 D). Post-menopause is characterized by a drop in estrogen levels because of the depletion of follicular oocytes that normally produce estrogen (Figure 3 D).

Noteworthy, aged mouse ovaries possess rare premeiotic germ cells that can generate oocytes following transplantation into a young host environment [65], and even further young adult donor bone marrow infusions into female mice postpone age-related reproductive failure [66]. In other words, some follicles may become unresponsive due to age-associated over-stimulation but can be rejuvenated.

Thus, reproductive aging is set in motion at puberty by an ever-increasing hypothalamic resistance to estrogens.  By increasing resistance of the hypothalamus to estrogens, the developmental program establishes the menstrual cycle at puberty. There is no program to cause menopause. It simply happens because resistance to estrogens (and some other hormones) is ever-increasing. This is an example of a quasi-program, a continuation of a program that was not switched off upon its completion (at puberty). The quasi-program interrupts the same reproductive function that the program establishes. The same mechanism (resistance of the hypothalamus to estrogen) first starts and then ends reproduction in women. An increased resistance to estrogens can explain both initiation and termination of the menstrual cycle.

How may we explain an increased resistance to estrogens? Resistance may be secondary to hyper-stimulation by estrogens themselves. In fact, in old acyclic mice, ovariectomy for 2 months partially reversed the hypothalamic resistance [41]. Hyper-stimulation of the hypothalamus by estrogens may cause resistance, in turn increasing stimulation of the ovary, until failure occurs. Alternatively, overstimulation of the hypothalamus with hormones and nutrients can cause estrogen-resistance. Is there a feedback resistance to overstimulation as shown in Figure 2? Then over-stimulation, with secondary resistance, is the driving cause of reproductive program and quasi-program. And most importantly over-stimulation occurs simultaneously both in the ovary and the brain.

## mTOR and menopause

**Figure 4.**
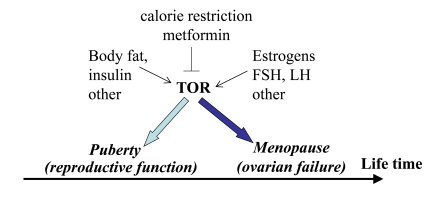
Program of puberty and quasi-program of menopause. The TOR pathway in the hypothalamus and the ovary is activated by growth factors, hormones (leptin, estrogens and FSH, LH, respectively) and nutrients. This activation starts menarche and then leads to menopause.

I propose that the increasing activation of mTOR (both in the hypothalamus and the ovary) drives hormone resistance, causing the onset of reproduction and then hyper-stimulation of the ovary and the hypothalamus and finally menopause (Figure 4).  Let us bring together several pieces of data.

First, mTOR is a regulator of puberty onset via modulation of the hypothalamus [67]. Also, both FSH and estrogens activate the mTOR pathway [68], [69]. So if TOR is activated constantly, it may not respond further to stimulation (hormone resistance).

Second, in mice lacking PTEN  in oocytes, the entire primordial follicle pool is activated. Subsequently, all primordial follicles become depleted in early adulthood, causing premature ovarian failure [70]. PTEN loss results in suppression of Foxo, so the Foxo was a primer suspect [70]. Yet, in theory loss of PTEN must also result in mTOR overactivation (Figure 1). I suggest that premature ovarian failure is caused by over-activation of TOR. (Note: this paper was initially written in 2008 and was ahead of its time and was not well received by conventional journals. Now it can be updated). It was shown tuberous sclerosis complex (Tsc), which negatively regulates mTOR, functions in oocytes to maintain the quiescence of primordial follicles. In mutant mice lacking the Tsc1 gene in oocytes, the entire pool of primordial follicles is activated prematurely due to elevated mTORC1 activity in the oocyte, ending up with follicular depletion in early adulthood and causing premature ovarian failure [71,72].

Third, calorie restriction (CR) prevents age-related increase in estrogen resistance in the hypothalamus of old female mice [73]. As already discussed, CR de-activates TOR [74]. I speculate that CR de-activates mTOR and delays estrogen resistance in the hypothalamus. Simultaneously, by deactivating mTOR in the oocytes, it may delay their depletion.

It was shown almost a century ago [75] and then reproduced numerous times that CR extends lifespan and prevents age-related infertility in rodents. In most of these studies, CR was initiated at weaning, causing a delayed onset of sexual maturation. So, the same condition (CR) delays both puberty and menopause. This is consistent with the notion that a quasi-program (menopause) is a mere continuation of the program (puberty).  But quasi-programs can be manipulated, exactly like programs. Recently it has been shown that a moderate caloric restriction initiated in rodents during adulthood sustains reproductive function of the female reproductive axis into advanced chronological age [76].

Fifth, metformin, an antidiabetic drug, activates AMPK and thus inhibits mTOR [77]. Furthermore, metformin inhibits mTOR in AMPK-independent manner too. Metformin restores ovulations in patients with premature menopause associated with polycystic ovary syndrome [56]. On the other hand, metformin delays a premature onset of the menstrual cycle [78]. So the same agent that inhibits the onset of reproductive function also inhibits its termination. This antagonistic pleiotropic effect is consistent with the notion of the same mechanism switching reproduction on and off. Metformin slowed down aging and the age-related switch-off of estrous function in mice [79]. Thus menopause can be delayed pharmacologically.

## Conclusion

This article presents two hypotheses. The first hypothesis explains (from both an evolutionary and mechanistic perspective) why aging is accelerated in men.  From the evolutionary perspective, the high accidental death rate in young men determines an accelerated aging. A model of TOR-driven aging provides a mechanistic explanation. When the accidental death rate is high, it is important to be bigger and stronger.  And the mTOR pathway is involved in growth and cellular hypertrophy. So, overactivated mTOR may be adaptive for young men.

But this can accelerate aging. At the cost of accelerated aging, over-stimulated mTOR pathway may provide an advantage earlier in life. And vice versa as discussed, "weak mTOR" provides disadvantage earlier in life and, vice versa, robustness and fast aging are associated [28]. Noteworthy, "competitive, aggressive personality" among men is associated with atherosclerosis and earlier death from age-related coronary disease [80].

The second hypothesis explains why menopause in women occurs despite slow-aging. Simply, the regulation of the menstrual cycle is fragile. There is a fine balance between ovarian stimulation by FSH and feedback hypothalamic responsiveness to estrogens. The menstrual cycle is vulnerable. Menopause is an example of a quasi-program (a program that was not switched off after its completion). In puberty, an increasing resistance to estrogen starts reproduction (a program). A further increase in the resistance (a quasi-program) causes overactivation of the ovary, decreasing fertility. This process can be treated pharmacologically (as any other age-related disease) to postpone menopause Potential therapeutic interventions to postpone menopause (as well as abolishment of the harmful consequences of menopause) will be discussed in forthcoming book The *Origin of Aging*.
